# Distal femoral osteotomy for the treatment of chronic patellofemoral instability improves gait patterns

**DOI:** 10.1007/s00402-025-05788-x

**Published:** 2025-03-08

**Authors:** Peter Rab, Romed P. Vieider, Lorenz Fritsch, Matthias Cotic, Florian B. Imhoff, Sebastian Siebenlist, Andrea Achtnich, Maximilian Hinz

**Affiliations:** 1 Department of Sports Orthopaedics, Technical University of Munich, Munich, Germany; 2 Department of Orthopaedics and Traumatology, University Hospital Basel, Basel, Switzerland

**Keywords:** Dynamic Q-angle, Gait pattern, Patellofemoral instability, Femoral antetorsion, Alignment, Distal femoral osteotomy

## Abstract

**Purpose:**

The purpose of this study was to evaluate pre- to postoperative changes in clinical and functional outcomes as well as gait patterns in patients who underwent surgery for chronic patellofemoral instability (PFI).

**Methods:**

Patients who underwent surgery for the treatment of recurrent PFI according to an individual risk factor analysis were included. Pre- and minimum 12 months postoperatively, patient-reported outcome measures (PROM; Kujala score, Lysholm score, Tegner Activity Scale [TAS] and Visual Analog Scale for pain) as well as gait (dynamic Q-angle) and function (dynamic valgus and dynamic Trendelenburg during single-leg squat) via videography were evaluated. Subgroup analysis was performed based on whether or not patients underwent concomitant distal femoral osteotomy (DFO) due to coronal and/or torsional malalignment.

**Results:**

Twenty-three patients were included (follow-up: 12.5 [12.1–13.0] months), of which 60.9% patients underwent a concomitant DFO. All PROM improved significantly (*p* < 0.05). Overall, dynamic Q-angle (*p* = 0.016) and dynamic valgus (*p* = 0.041) were observed significantly less frequently postoperatively when to compared to preoperatively. Subgroup analysis showed that only the group that underwent DFO had a significant improvement of dynamic Q-angle (*p* = 0.041). Dynamic Trendelenburg did not improve (*p* > 0.05). Regression analysis showed that the presence of a postoperative dynamic Q-angle was associated with a worse postoperative Kujala score (*p* = 0.042) and TAS (*p* = 0.049).

**Conclusion:**

Patient-individualized surgery for PFI improved gait patterns and functional testing, especially in patients who also underwent DFO. The presence of dynamic Q-angle postoperatively was associated with significantly worse functional outcome and sporting ability.

**Level of evidence:**

Level III.

## Introduction

Patellofemoral instability (PFI) is a multifactorial pathology that mostly affects young female patients [1]. If left untreated, it may lead to persistent instability, pain, impaired quality of life and early posttraumatic osteoarthritis [2, 3, 4].

Multiple static and dynamic stabilizers contribute to patellofemoral stability [5, 6]. In patients with chronic PFI, however, this complex system is disrupted which may lead to altered lower extremity kinematics and gait abnormalities such as, reduced gait speed, increased valgus moment and/or increased internal-rotation-adduction moment - a so-called dynamic Q-angle [7, 8, 9, 10]. These abnormalities may be amplified in patients with increased femoral antetorsion (femAT), which has also been identified as a risk factor for PFI itself [8, 11] as well as instability recurrence if left untreated [12, 13, 14]. For patients with PFI and increased femAT and/or valgus malalignment, derotational and/or varus-producing distal femoral osteotomy (DFO) may be a suitable treatment option with favorable outcomes and low reported rates of instability recurrence [15, 15, 16, 17, 18, 19, 20, 21, 22, 23]. While some studies have reported favorable outcomes following isolated medial patellofemoral ligament (MPFL) reconstruction regardless of elevated femAT, other authors have observed inferior outcomes in patients with increased femAT who underwent isolated MPFL reconstruction compared to those who received concomitant DFO [14, 24, 25]. Therefore, failure to treat elevated femAT may have a negative impact on the postoperative outcome.

Although changes in postoperative gait patterns have been reported for patients who underwent surgery for PFI without alignment-correcting procedures [26, 27, 28], comparable data for patients with malalignment who underwent DFO are scarce.

Therefore, the purpose of the present study was to evaluate pre- to postoperative changes in gait patterns and functional strength in patients who underwent surgery for PFI with or without alignment-correcting DFO. It was hypothesized that the gait pattern and functional strength would improve postoperatively, but that changes during gait would predominantly be observed in patients with concomitant DFO. Further, the postoperative presence of a pathological gait pattern would correlate with inferior outcomes.

## Materials and methods

This prospective clinical study was approved by the institutional review board of the Technical University of Munich (reference number: 17/18S) and conducted according to the Declaration of Helsinki. All patients provided their written and informed consent.

Patients with recurrent PFI (≥ 2 patellar subluxations or dislocations) who were scheduled for surgery in a single institution between March 2019 and January 2020 were prospectively enrolled. Surgical treatment was tailored based on an individual risk factor analysis and included reconstruction of the MPFL, tibial tubercle osteotomy (TTO), alignment-correcting osteotomy (specifically defined as derotational DFO, varus-producing DFO or high tibial osteotomy), trochleoplasty and treatment of (osteo-)chondral lesions. Further, only patients that had already participated in a pilot study, during which preoperative gait and functional strength of patients with PFI were compared with healthy controls, were considered for inclusion [8]. Patients with open growth plates, severe pain and disability during walking without crutches, an acute traumatic patellar dislocation and those with previous lower limb surgery, severe trauma, musculoskeletal disorders or planned arthroplasty were excluded.

### Preoperative assessment and surgical planning

All patients underwent a standardized clinical and radiological assessment to evaluate the underlying risk factors for PFI. Physical examination was conducted to assess patellar (mal)tracking, apprehension and range of motion of the knee and hip. Weight-bearing whole-leg anteroposterior radiographs were performed to evaluate coronal limb alignment, including femorotibial angle, mechanical lateral distal femoral angle, and mechanical medial proximal tibial angle [29]. In patients with coronal malalignment, preoperative planning was performed using a digital planning software (mediCAD® version 5.1, Hectec, Altdorf, Germany). Patellar height was evaluated on lateral knee radiographs using the Caton-Deschamps index (CDI) [30]. Trochlear dysplasia was classified according to Dejour [31]. Further, lower extremity magnetic resonance imaging was conducted to obtain femAT, patellar tilt, tibial tuberosity-trochlear groove (TT-TG) distance and trochlear dysplasia. Femoral antetorsion was measured as described by Schneider et al. [32].

Derotational DFO was performed in cases with increased femAT. The cut-off value for increased femAT varied between > 15° and > 20° as different surgeons were involved in clinical practice, and the threshold was lowered if a concomitant valgus deformity indicating surgical correction was observed [18, 33]. A varus-producing, lateral opening-wedge DFO was performed where valgus malalignment was present. The cut-off value was typically ≥ 3°, but this may have been lower in patients in which a concomitant correction of increased femAT was indicated [3]. In cases with increased femAT and valgus deformity, derotational and valgus-correcting DFO was indicated simultaneously, as described previously [18]. Tibial tubercle osteotomy was indicated in patients with a lateralized tibial tuberosity (TT-TG distance > 20 mm) or patella alta (CDI ≥ 1.2). Trochleoplasty was performed in patients with a positive J-sign, apprehension at 60° of knee flexion and trochlear dysplasia Type B/D on lateral radiographs. Reconstruction of the MPFL was indicated in cases of high lateralization tendency following the abovementioned procedures or isolated when anatomic risk factors were absent.

### Surgical technique

In all cases, diagnostic arthroscopy was performed to assess the cartilage, patellar tracking, and the medial retinaculum complex. Biplanar derotational DFO with an anterior closing wedge was performed via a standardized lateral subvastus approach as previously reported by the authors [18, 34]. Additional correction of valgus deformity was performed concomitantly by adding a lateral opening wedge with the aim of neutral alignment as previously described [18, 34]. Biplanar lateral open-wedge DFO was performed to treat isolated valgus malalignment without increased femAT as previously described with the aim of neutral alignment [35]. To secure the osteotomy, an internal plate fixation system with locking screws was used (Tomofix distal femoral plate, DePuy Synthes, Umkirch, Germany). Tibial tubercle osteotomy was performed as described by Fulkerson [36]. Trochleoplasty was performed as described by Bereiter [37, 38]. Reconstruction of the MPFL was performed using the ipsilateral gracilis tendon as described by Schöttle et al. [39].

### Postoperative rehabilitation

In cases of isolated MPFL reconstruction, weight-bearing was limited to 20 kg for 2 weeks. Patients who underwent DFO and/or trochleoplasty alone or in combination with other procedures were restricted to 6 weeks of partial weight-bearing. Weight-bearing was then gradually increased. Physical therapy was started on the first postoperative day, including passive mobilization and gait training with crutches. Therapy was then continued 2–3 times a week,with active exercises added according to the surgery performed and the weight bearing permitted. In the case of MPFL reconstruction, flexion was limited to 90° for 6 weeks. Range of motion was limited to 60° of flexion and 20° of extension for 2 weeks and gradually increased thereafter in patients who underwent trochleoplasty. For isolated DFO, range of motion was not limited postoperatively. For patients who underwent TTO, flexion was limited to 20° for 2 weeks and then gradually increased.

### Clinical and functional outcome assessment

Pre- and minimum 12 months postoperatively, patient-reported outcome measures (PROM) were obtained and clinical examination as well as gait videography were performed. Assessment of PROM included the Kujala score, Lysholm score, Tegner Activity Scale (TAS) and Visual Analog Scale (VAS) for pain at rest and during activity. A goniometer was used to measure bilateral passive hip internal rotation (hipIR), hip external rotation (hipER) [40], static Q-angle and tubercle-sulcus angle (TS angle) [41, 42]. Further, rates of postoperative patellar re-dislocation and complications that required revision surgery were collected. Patients who underwent revision surgery were excluded from all analyses.

### Gait and single-leg squat analyses

**Fig. 1:**
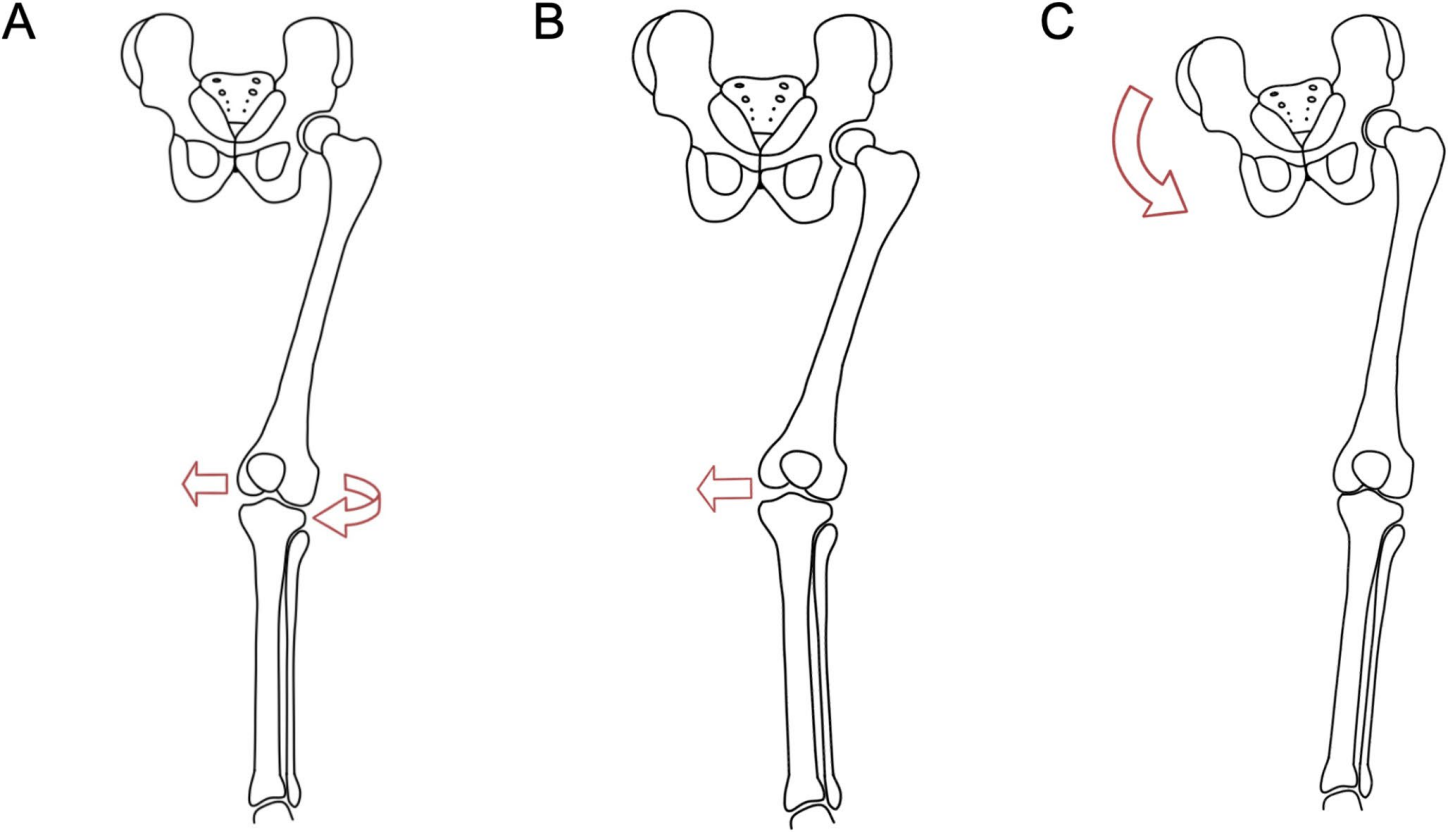
Gait patterns defined for frontal plane gait analysis. **A**, dynamic Q-angle, defined as an obvious internal-rotation-adduction moment of the knee while walking; **B**, dynamic valgus during a single-leg squat; **C**, dynamic Trendelenburg, defined as pelvic tilting > 10° or cork-screwing during a single leg squat

Gait analysis was performed in a standardized manner as previously described, see Fig. 1 [8]. Five gait cycles of normal walking were recorded and repeated three times. Gait cycles were recorded using optical markers on anatomical landmarks (anterior superior iliac spine, center of the patella and tibial tuberosity) [43]. During walking, the presence of a dynamic Q-angle, defined as an obvious internal-rotation-adduction moment of the knee, was analyzed.

Further, single-leg squats were performed bilaterally at approximately 60° of knee flexion over a 5-second period. The presence of a dynamic valgus during the single-leg squat, defined as a medial deviation of the center of the knee on an imaginary line between the center of the hip to the center of the ankle [44], and/or a dynamic Trendelenburg, defined as pelvic tilting > 10° or cork-screwing during a single leg squat, were evaluated [45, 46]. The single-leg squat was not included in the analysis if it was not possible for the patient to perform a single-leg squat due to limited range of motion, apprehension and/or pain.

Recordings were obtained as frontal view videography via a steady mirrorless digital camera (camera: FUJIFILM X-T20, Fujifilm Holdings Corporation, Japan; lens: FUJINON XF16-55 mm F2.8, Fujifilm Holdings Corporation, Japan) mounted at a height of 80 cm above floor level and perpendicular to the walking path. Post-processing was performed via Final Cut Pro (Apple Inc., USA). All videos were analyzed by three independent observers.

### Statistical analysis

Statistical analysis was performed using RStudio (RStudio Public Benefit Corporation, Boston, USA) and R version 4.2.3 (R Foundation for Statistical Computing, Vienna, Austria). Normality assessment of the data was conducted using the Shapiro-Wilk test. Mean ± SD was used to report normally distributed data whereas median (interquartile range) was used to report non-normally distributed data. The paired t-test was applied to compare normally distributed data and the Wilcoxon signed rank test was used for non-normally distributed data. McNemar’s test was employed to compare the incidence of gait patterns across time points. Logistic regression analysis was performed to assess the relationship between the presence of postoperative dynamic gait patterns and clinical and functional outcome measurements. As this study was performed as a continuation of a prior study, an additional power analysis was not conducted [8]. Interrater reliability among three observers was assessed using Light’s kappa [47] and interpreted according to Landis and Koch (≤ 0.20: slight agreement, 0.21 to 0.40: fair agreement, 0.41 to 0.60: moderate agreement, 0.61 to 0.80: substantial agreement, and 0.81 to 1.0: almost perfect or perfect agreement) [48]. A significance level of < 0.05 was chosen, and all *p*-values were two-tailed.

## Results

Of 35 patients that participated in the initial study, three patients (8.6%) underwent revision surgery during follow-up and were excluded (1x valgus deformity after derotational DFO, 1x infected pseudarthrosis following valgus-correcting DFO, 1x delayed union after derotational DFO).

**Fig. 2:**
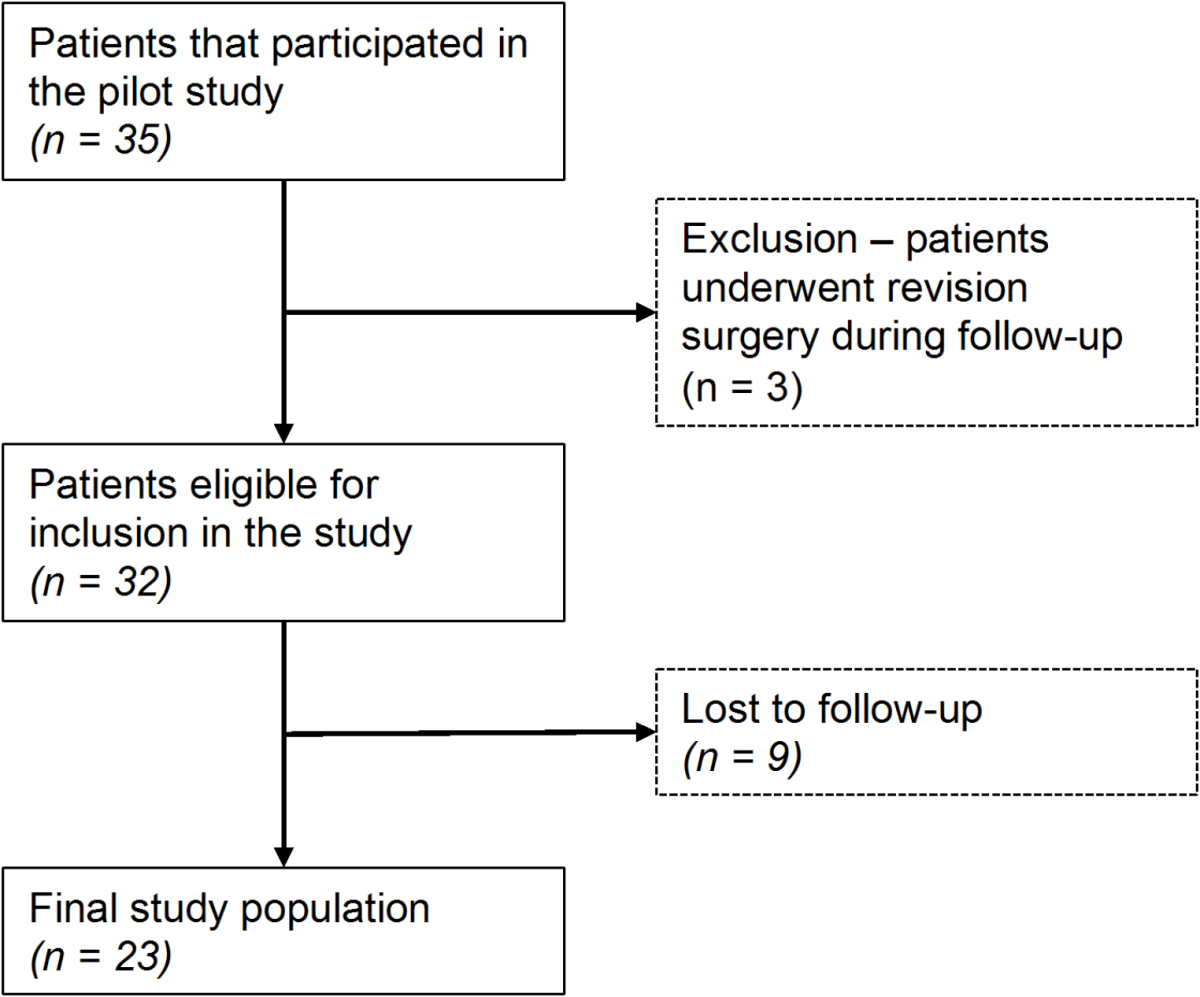
Flowchart of patient inclusion

**Fig. 3:**
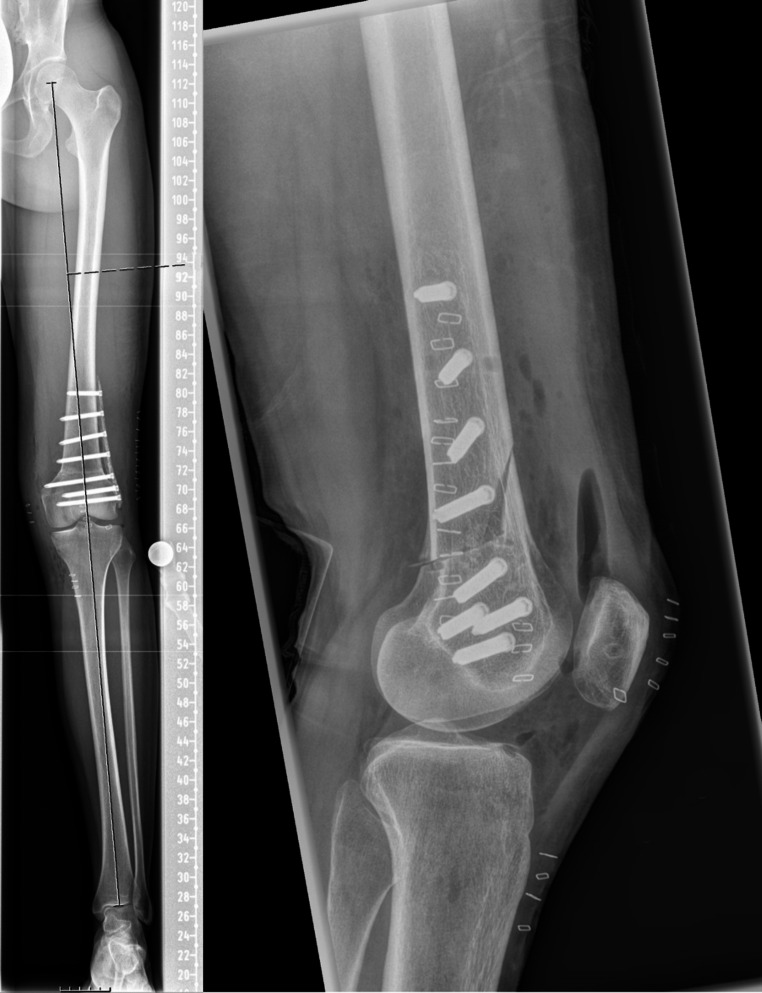
Postoperative whole-leg and lateral radiograph of concomitant derotational and varus-producing biplanar distal femoral osteotomy

Of the remaining patients, 23 (71.9% follow-up) were included in the present study at a median postoperative follow-up of 12.5 (12.1–13.0) months, see Fig. 2. The majority of patients (60.9%) underwent a concomitant DFO, see Fig. 3. None of the patients suffered a patellar re-dislocation during follow-up. Details on patient demographics and the procedures performed are reported in Table 1.

**Table 1:** Patient demographics and performed procedures. BMI, body mass index; DFO, distal femoral osteotomy; TTO, tibial tubercle osteotomy; MPFL, medial patellofemoral ligament

Patient demographics	
Age at surgery, years ± standard deviation	23.7 ± 6.9
Sex, female: male (% female)	19:4 (82.6)
BMI, kg/m^2^ ± standard deviation	32.1 ± 3.2
Procedures performed	*N* (%)
With DFO, n (%)	14 (60.9)
Varus-producing DFO	6 (26.1)
With concomitant procedures	5 (21.7)
MPFL reconstruction	2 (8.7)
Trochleoplasty	1 (4.3)
TTO	1 (4.3)
Trochleoplasty and MPFL reconstruction	1 (4.3)
Isolated	1 (4.3)
Isolated derotational DFO	5 (21.7)
Combined derotational and varus-producing DFO	3 (13.0)
Isolated	2 (8.7)
With MPFL reconstruction	1 (4.3)
Without DFO, n (%)	9 (39.1)
Isolated MPFL reconstruction	8 (34.8)
Trochleoplasty and MPFL reconstruction	1 (4.3)

### Clinical and functional outcome

**Fig. 4:**
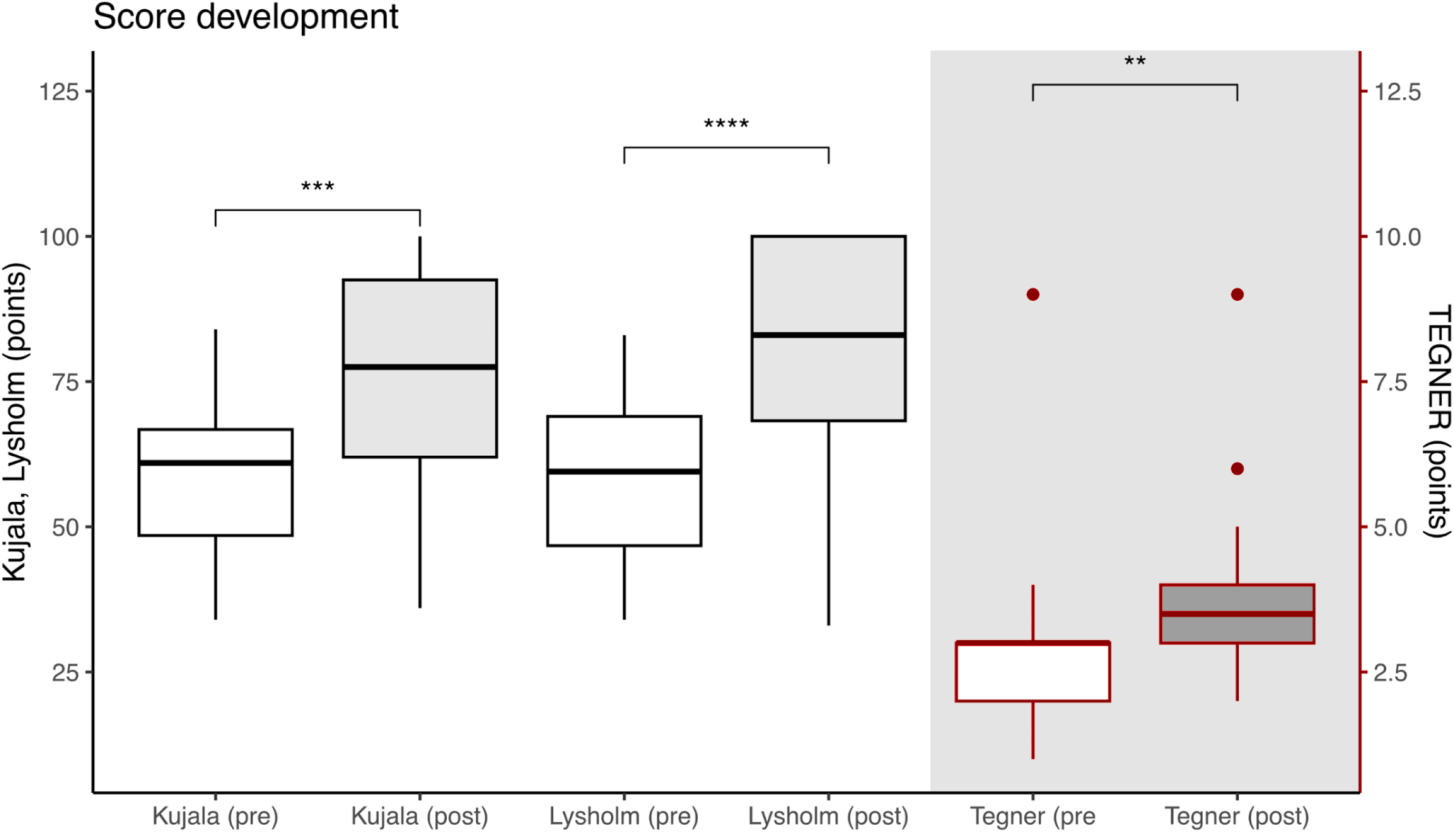
Functional outcome parameters at pre- and postoperative time point. * *p* < 0.05. ** *p* < 0.01. *** *p* < 0.001. **** *p* < 0.0001. Pre: pre-operative. Post: post-operative

From pre- to postoperatively, knee function, sporting ability and pain improved significantly, see Table 2; Fig. 4. Further, patients who underwent an alignment-correcting osteotomy exhibited a significant reduction in the static Q-angle (14 [12, 13, 14, 15, 16, 17, 18, 19, 20] ° preoperatively vs. 11 [10, 11, 12] ° postoperatively, *p* = 0.031), whereas no significant change was observed in patients who did not undergo an alignment-correcting osteotomy (*p* > 0.05). Tubercle-sulcus angle was significantly reduced postoperatively (*p* = 0.001). Passive hipIR decreased (*p* < 0.001) whereas passive hipER did not change significantly (*p* > 0.05). Data on PROM, static Q-angle, TS angle and hip range of motion are reported in Tables 2 and 3. No significant difference was observed in VAS (*p* = 1), Kujala Score (*p* = 0.088) and TAS (*p* = 0.11) between patients who underwent DFO and those who did not. Patients who underwent DFO had a significantly lower Lysholm score than patients without DFO (DFO: 77 [65–84], no DFO: 100 [95–100], *p* = 0.049).

**Table 2:** Pre- and postoperative clinical and functional outcome and preoperative radiographic measurements. Significant *p*-values are bolded. HipER, hip external rotation; HipIR, hip internal rotation; mLDFA, mechanical lateral distal femoral angle; mMPTA, mechanical medial proximal tibial angle; TAS, Tegner activity scale; TS, tubercle-sulcus; VAS, visual analog scale, N.s., not significant

	Preoperative	Postoperative	*p*-value
Clinical and functional outcome			
VAS for pain at rest			
Overall (*n* = 23)	2 (0–4)	0 (0–1)	**0.005**
Patients with DFO (*n* = 14)	2 (0-3.5)	0 (0–1)	n.s.
Patients without DFO (*n* = 9)	4 (2–5)	0 (0–1)	0.28
VAS for pain during activity			
Overall	5 (4.5–7)	2 (0.5-6)	**0.011**
Patients with DFO	5 (4–7)	3 (1–6)	n.s.
Patients without DFO	6 (5–7)	2 (0–6)	0.28
Kujala score			
Overall	57.9 ± 13.2	76.5 ± 18.0	**< 0.001**
Patients with DFO	56.6 ± 10.9	70.7 ± 14.3	**0.007**
Patients without DFO	59.8 ± 16.6	85.2 ± 20.4	**0.01**
TAS			
Overall	3.0 (2.0–3.0)	4.0 (3.0–4.0)	**0.002**
Patients with DFO	3.0 (2.0–3.0)	3.0 (3.0–4.0)	n.s.
Patients without DFO	3.0 (2.0–3.0)	4.0 (4.0–5.0)	n.s.
Lysholm score			
Overall	58 (48–68)	81 (69–100)	**< 0.001**
Patients with DFO	62 (51–59)	77 (65–84)	**0.018**
Patients without DFO	55 (46–67)	100 (95–100)	**0.009**
Preoperative radiographic measurements			
Femorotibial axis, °			
Overall	2.8 (1.6–3.3)		
Patients with DFO	3.2 (2.2–4.5)		
Patients without DFO	1.9 (1.5–2.6)		
mLDFA, °			
Overall	87.5 (86.2–88.7)		
Patients with DFO	87.4 (86.3–88.7)		
Patients without DFO	87.5 (86.1–88.5)		
mMPTA, °			
Overall	90.5 (88.6–90.9)		
Patients with DFO	90.0 (88.1–91.0)		
Patients without DFO	90.7 (90.3–90.8)		
Patellar tilt, °			
Overall	19.5 (12.2–24.1)		
Patients with DFO	19.9 (17.5–28.5)		
Patients without DFO	15.4 (12.1–23.8)		

### Gait and single-leg squat analysis

Overall, there was a significant reduction in the presence of dynamic Q-angle during gait (69.6% preoperatively vs. 30.4% postoperatively, *p* = 0.016) Notably, this reduction in dynamic Q-angle was significant in the patient group that underwent an alignment-correcting osteotomy (64.3% preoperatively vs. 21.4% postoperatively, *p* = 0.041) but not in patients who did not undergo an alignment-correcting osteotomy (77.8% preoperatively vs. 44.4% postoperatively, *p* > 0.05). Overall, dynamic valgus during single-leg squat improved significantly (65.2% preoperatively vs. 39.1% postoperatively, *p* = 0.041), but was not significant when the subgroups were analyzed separately (*p* > 0.05). Dynamic Trendelenburg did not decrease postoperatively (*p* > 0.05).

Interrater reliability was “fair” (dynamic Q-angle during [interrater kappa: 0.30], dynamic valgus [interrater kappa: 0.38]) to “moderate” (dynamic Trendelenburg [interrater kappa: 0.41]).

**Table 3:** Incidence of gait patterns at preoperative and postoperative time points. Significant *p-*values are bolded. HipER, hip external rotation; HipIR, hip internal rotation; N.s., not significant; TS, tubercle-sulcus

	Pre-operative	Post-operative	*p*-value
Dynamic Q-angle during gait, n (%)			
Ipsilateral			
Overall (*n* = 23)	16 (69.6%)	7 (30.4%)	**0.016**
Patients with DFO (*n* = 14)	9 (64.3%)	3 (21.4%)	**0.041**
Isolated derotational DFO (*n* = 3)	2 (66.7%)	0 (0%)	
Isolated varus-producing DFO (*n* = 5)	5 (100%)	2 (40%)	
Combined DFO (*n* = 6)	3 (50%)	1 (16.7%)	
Patients without DFO (*n* = 9)	7 (77.8%)	4 (44.4%)	n.s
Contralateral	12 (52.2%)	7 (30.4%)	n.s.
Dynamic valgus during single-leg squat, n (%)			
Ipsilateral			
Overall	15 (65.2%)	9 (39.1%)	**0.041**
Patients with DFO	8 (57.1%)	4 (28.5%)	n.s.
Patients without DFO	7 (77.8%)	5 (55.6%)	n.s.
Contralateral	17 (73.9%)	13 (56.5%)	n.s.
Dynamic Trendelenburg during single-leg squat, n (%)			
Ipsilateral			
Overall	9 (39.1%)	8 (34.8%)	n.s.
Patients with DFO	6 (42.9%)	6 (42.9%)	n.s.
Patients without DFO	3 (33.3%)	2 (22.2%)	n.s.
Contralateral	13 (56.5%)	7 (30.4%)	n.s.
Static Q-angle, deg			
Ipsilateral			
Overall	13 (11–18)	11 (10–12)	**0.026**
Patients with DFO	14 (12–20)	12 (12–14)	**0.031**
Patients without DFO	11 (10–15)	10 (10–11)	n.s.
Contralateral	12 (10–16)	11 (10–15])	n.s.
TS angle, deg			
Ipsilateral			
Overall	10 (8–12)	5 (3–9)	**0.001**
Patients with DFO	10 (8–11)	7 (5–9)	**0.005**
Patients without DFO	12 (8–12)	3 (3–9)	**0.040**
Contralateral	8 (8–10)	5 (3–9)	0.004
HipIR, deg			
Ipsilateral			
Overall	46 ± 12	36 ± 7	**< 0.001**
Patients with DFO	45 ± 7	37 ± 8	**0.010**
Patients without DFO	50 ± 18	34 ± 6	**0.030**
Contralateral	47 ± 12	38 ± 8	**< 0.001**
HipER, deg			
Ipsilateral			
Overall	35 (27–50)	38 (32–41)	n.s.
Patients with DFO	41 (31–50)	39 (33–43)	n.s.
Patients without DFO	29 (20–37)	35 (32–40)	n.s.
Contralateral	40 (28–45)	38 (30–40)	n.s.

### Correlation between postoperative gait and single-leg squat and functional outcome

Patients with a postoperative dynamic Q-angle gait pattern had a significantly lower Kujala score than patients without, see Table 4. Further, logistic regression analysis showed a significant influence of the presence of a postoperative dynamic Q-angle on the Kujala score (*p* = 0.042) and TAS (*p* = 0.049), but not on the Lysholm score (*p* > 0.05). Additionally, dynamic valgus during single-leg squat had a significant negative impact on the Kujala score (*p* = 0.021), but not on the Lysholm score (*p* > 0.05) or the TAS (*p* > 0.05). Dynamic Trendelenburg did not influence the Kujala score (*p* > 0.05), TAS (*p* > 0.05) or the Lysholm score (*p* > 0.05).

**Table 4:** Patient-reported outcome measures according to the incidence of postoperative gait patterns and functional tests. Significant *p*-values are bolded. N.s., not significant

	Kujala score	Tegner Activity Scale	Lysholm score
Postoperative dynamic Q-angle during gait			
Absent	72 ± 16	4 (3–4)	86 (73–100)
Present	46 ± 11	4 (3–6)	75 (50–95)
*p*-value	**0.041**	n.s.	n.s.
Postoperative dynamic valgus during single-leg squat			
Absent	78 ± 19	4 (3–4)	90 (79–100)
Present	60 ± 23	3 (3–5)	69 (53–95)
*p*-value	n.s.	n.s.	n.s.
Dynamic Trendelenburg during single-leg squat			
Absent	80 ± 14	4 [3–4]	93 [74–100]
Present	63 ± 19	3 [3–3]	73 [68–81]
*p*-value	n.s.	n.s.	n.s.

## Discussion

The most important findings of the present study were that patients who underwent patient-individualized surgery for PFI had a significant improvement of knee function, sporting ability and pain. Overall, a significant reduction of static Q-angle, lower incidence of dynamic Q-angle during gait and dynamic valgus during single-leg squat were observed. The findings regarding gait and single-leg squat were statistically significant for the overall cohort (dynamic Q-angle during gait, dynamic valgus during single-leg squat) and for patients with alignment-correcting osteotomies (static Q-angle and dynamic Q-angle during gait), but not for patients without alignment-correcting osteotomies. It should be noted that the presence of a dynamic Q-angle during gait and dynamic valgus during single-leg squat was associated with significantly inferior postoperative knee function and sporting ability.

Few studies have previously investigated postoperative gait patterns in patients with PFI [26, 27, 28]. Carnesecchi et al. [28] reported that patients who underwent MPFL reconstruction showed a postoperative gait pattern that was similar to healthy controls during normal and fast walking speeds. Notably, gait abnormalities were observed in these patients during running. The degree of improvement, however, could not be assessed as the patients in their cohort were only assessed postoperatively. The gait pattern of patients who underwent MPFL reconstruction improved to a comparable degree in the present study without reaching statistical significance, potentially due to the small sample size. Ammann et al. [26] reported that patients who underwent bilateral trochleoplasty for bilateral PFI showed gait patterns similar to healthy controls. Patients with unilateral PFI who underwent unilateral trochleoplasty, however, still showed a pathological gait pattern postoperatively. Their observations may indicate that chronic PFI may be considered a bilateral pathology even in patients with unilateral instability. To the knowledge of the authors of the present study, the present study was the first study to conduct a pre- and postoperative gait analysis and functional assessment in patients with PFI whereby patients undergoing alignment-correcting procedures were also included.

When patients with vs. without alignment-correction were analyzed separately, only the group that underwent a concomitant alignment-correcting procedure showed a significant improvement of dynamic Q-angle. This may, in part, be related to the close relation between the presence of increased femAT and compensatory gait mechanics, which has also been observed in previous studies [8, 10, 49, 50]. A possible explanation for this observation may be a compensation for an altered abduction moment arm of the hip which may lead to increased mediolateral contact forces in the patellofemoral joint [10, 49]. Further, patients who underwent a DFO also had a significant decrease in static Q-angle. This has been shown previously, specifically in the context of varization [51]. This decrease in static Q-angle may lead to a decrease in dynamic Q-angle which may further explain why patients who did not undergo an osteotomy did not have a significant change in dynamic Q-angle. It should be noted that other factors, including muscular, neurological and psychological, may influence gait as well [7, 9, 10, 27, 52, 53]. This may explain why patients who did not require alignment-correction also had pathological gait patterns, why a physiological gait pattern was not observed in all patients with an alignment-correction postoperatively, and why dynamic Trendelenburg, as a highly demanding test, did not improve. Consequently, in patients with malalignment, specifically increased femAT, alignment-correction should be considered as it may improve not only functional outcome, but also gait. When an alignment-correction is not performed in these patients, inferior outcomes may be expected as the presence of a postoperative dynamic Q-angle was associated with inferior outcomes. This aligns with the findings of previous studies that showed that when an alignment-correction is not performed in patients with increased femAT, inferior clinical and functional outcomes may be expected [11, 13, 14, 23, 25].

As a result, the frontal plane gait analysis performed in this study, particularly the dynamic Q-angle gait pattern, should be incorporated into daily clinical practice as a simpler, more accessible alternative to 3D gait analysis that allows for timely assessment. While clinical evaluations have demonstrated inconsistency in determining the indication for (derotational) DFO, this gait pattern could serve as a valuable tool for identifying patients who would benefit from alignment-correcting osteotomy. However, because alignment-correcting osteotomy was often combined with other osseous and soft tissue procedures in this study, the effect of DFO alone on postoperative gait improvement could not be assessed fully.

Several limitations should be considered when interpreting the results of the present study. First, the patients included in the pilot study represented a heterogenous cohort including different pathogenetic factors that may have caused PFI and consequently, led to different surgical procedures being performed. This does however represent clinical practice when treating patients with PFI. Further, although only patients who underwent a DFO showed a significant reduction in dynamic Q-angle, this would likely also have been significant in patients without DFO if the sample size were larger as a trend for a reduction was observed in these patients. Additionally, the interrater analysis conducted by this study showed a lower interrater reliability than in the pilot study [8]. This difference may be attributed to decreased postoperative femAT values which may have made the gait and functional analysis more challenging. This observation was also reflected in our pilot study where more extreme values of femAT correlated with higher interobserver agreement [8]. A more detailed gait analysis, including a 3-dimensional visualization of gait patterns, could have provided more information regarding changes in gait and may result in higher interrater reliability. In the pilot study as well as the present study, the gait and function-related outcomes were kept binary as means to improve simplicity and consequently, its applicability during clinical practice.

## Conclusion

Patient-individualized surgery for PFI improved gait patterns and functional testing, especially in patients who also underwent DFO. The presence of dynamic Q-angle postoperatively was associated with significant worse functional outcome and sporting ability.

## Data Availability

No datasets were generated or analysed during the current study.

## Abbreviations

BMI: Body mass index
DFO: Distal femoral osteotomy
femAT: Femoral antetorsion
mLDFA: Mechanical lateral distal femoral angle
mMPTA: Medial proximal tibial angle
MPFL: Medial patellofemoral ligament
TAS: Tegner Activity Scale
TS: Tubercle-sulcus angle
TTO: Tibial tubercle osteotomy
TT-TG: Tibial tubercle-trochlear groove
VAS: Visual Analog Scale

## References

[1] Zheng ET, Kocher MS, Wilson BR et al (2022) Descriptive epidemiology of a surgical patellofemoral instability population of 492 patients. Orthop J Sports Med 10. 10.1177/23259671221108174 10.1177/23259671221108174 PMC9289910 35859643

[2] Arnbjornsson A, Egund N, Rydling O et al (1992) The natural history of recurrent dislocation of the patella. Long-term results of Conservative and operative treatment. J Bone Joint Surg Br 74–B:140–142. 10.1302/0301-620X.74B1.1732244 10.1302/0301-620X.74B1.1732244 1732244

[3] Hinz M, Weyer M, Brunner M et al (2024) Varus osteotomy as a salvage procedure for young patients with symptomatic patellofemoral arthritis and valgus malalignment at short- to mid-term follow-up: a case series. Arch Orthop Trauma Surg 144:1667–1673. 10.1007/s00402-024-05212-w 38386061 10.1007/s00402-024-05212-w PMC10965738

[4] Magnussen RA, Verlage M, Stock E et al (2017) Primary patellar dislocations without surgical stabilization or recurrence: how well are these patients really doing? Knee Surg Sports Traumatol Arthrosc 25:2352–2356. 10.1007/s00167-015-3716-3 26215775 10.1007/s00167-015-3716-3

[5] Siljander B, Tompkins M, Martinez-Cano JP (2022) A review of the lateral patellofemoral joint: anatomy, biomechanics, and surgical procedures. JAAOS Glob Res Rev 6. 10.5435/JAAOSGlobal-D-21-00255 10.5435/JAAOSGlobal-D-21-00255 PMC9302287 35858252

[6] Watts RE, Gorbachova T, Fritz RC et al (2023) Patellar tracking: an old problem with new insights. Radiographics 43:e220177. 10.1148/rg.220177 37261964 10.1148/rg.220177 PMC10262599

[7] Camathias C, Ammann E, Meier RL et al (2020) Recurrent patellar dislocations in adolescents result in decreased knee flexion during the entire gait cycle. Knee Surg Sports Traumatol Arthrosc 28:2053–2066. 10.1007/s00167-020-05911-y 32130443 10.1007/s00167-020-05911-y

[8] Imhoff FB, Cotic M, Dyrna FGE et al (2021) Dynamic Q-angle is increased in patients with chronic patellofemoral instability and correlates positively with femoral torsion. Knee Surg Sports Traumatol Arthrosc 29:1224–1231. 10.1007/s00167-020-06163-6 32683477 10.1007/s00167-020-06163-6

[9] Lucas KCH, Jacobs C, Lattermann C, Noehren B (2020) Gait deviations and muscle strength deficits in subjects with patellar instability. Knee 27:1285–1290. 10.1016/j.knee.2020.05.008 32591208 10.1016/j.knee.2020.05.008

[10] Passmore E, Graham HK, Pandy MG, Sangeux M (2018) Hip- and patellofemoral-joint loading during gait are increased in children with idiopathic torsional deformities. Gait Posture 63:228–235. 10.1016/j.gaitpost.2018.05.003 29775910 10.1016/j.gaitpost.2018.05.003

[11] Kaiser P, Schmoelz W, Schoettle P et al (2017) Increased internal femoral torsion can be regarded as a risk factor for patellar instability - a biomechanical study. Clin Biomech Bristol Avon 47:103–109. 10.1016/j.clinbiomech.2017.06.007 10.1016/j.clinbiomech.2017.06.007 28628800

[12] Franciozi CE, Ambra LF, Albertoni LJB et al (2019) Anteromedial tibial tubercle osteotomy improves results of medial patellofemoral ligament reconstruction for recurrent patellar instability in patients with tibial tuberosity-trochlear groove distance of 17 to 20 mm. Arthrosc J Arthrosc Relat Surg Off Publ Arthrosc Assoc N Am Int Arthrosc Assoc 35:566–574. 10.1016/j.arthro.2018.10.109 10.1016/j.arthro.2018.10.109 30612771

[13] Kaiser P, Schmoelz W, Schöttle PB et al (2019) Isolated medial patellofemoral ligament reconstruction for patella instability is insufficient for higher degrees of internal femoral torsion. Knee Surg Sports Traumatol Arthrosc Off J ESSKA 27:758–765. 10.1007/s00167-018-5065-5 10.1007/s00167-018-5065-5 30062643

[14] Zhang Z, Zhang H, Song G et al (2020) Increased femoral anteversion is associated with inferior clinical outcomes after MPFL reconstruction and combined tibial tubercle osteotomy for the treatment of recurrent patellar instability. Knee Surg Sports Traumatol Arthrosc Off J ESSKA 28:2261–2269. 10.1007/s00167-019-05818-3 10.1007/s00167-019-05818-3 31797022

[15] Deng X, Li L, Zhou P et al (2021) Medial patellofemoral ligament reconstruction combined with biplanar supracondylar femoral derotation osteotomy in recurrent patellar dislocation with increased femoral internal torsion and genu valgum: a retrospective pilot study. BMC Musculoskelet Disord 22:990. 10.1186/s12891-021-04816-2 34836529 10.1186/s12891-021-04816-2 PMC8626929

[16] Frings J, Krause M, Akoto R, Frosch K-H (2019) Clinical results after combined distal femoral osteotomy in patients with patellar maltracking and recurrent dislocations. J Knee Surg 32:924–933. 10.1055/s-0038-1672125 30282099 10.1055/s-0038-1672125

[17] Frings J, Krause M, Akoto R et al (2018) Combined distal femoral osteotomy (DFO) in genu valgum leads to reliable patellar stabilization and an improvement in knee function. Knee Surg Sports Traumatol Arthrosc Off J ESSKA 26:3572–3581. 10.1007/s00167-018-5000-9 10.1007/s00167-018-5000-9 29869201

[18] Hinz M, Cotic M, Diermeier T et al (2023) Derotational distal femoral osteotomy for patients with recurrent patellar instability and increased femoral antetorsion improves knee function and adequately treats both torsional and valgus malalignment. Knee Surg Sports Traumatol Arthrosc 31:3091–3097. 10.1007/s00167-022-07150-9 36109379 10.1007/s00167-022-07150-9 PMC10356631

[19] Imhoff FB, Beitzel K, Zakko P et al (2018) Derotational osteotomy of the distal femur for the treatment of patellofemoral instability simultaneously leads to the correction of frontal alignment: A laboratory cadaveric study. Orthop J Sports Med 6:2325967118775664. 10.1177/2325967118775664 29900182 10.1177/2325967118775664 PMC5985607

[20] Klasan A, Compagnoni R, Grassi A, Menetrey J (2024) Promising results following derotational femoral osteotomy in patellofemoral instability with increased femoral anteversion: A systematic review on current indications, outcomes and complication rate. J Exp Orthop 11:e12032. 10.1002/jeo2.12032 38774579 10.1002/jeo2.12032 PMC11106799

[21] Nha KW, Ha Y, Oh S et al (2018) Surgical treatment with closing-wedge distal femoral osteotomy for recurrent patellar dislocation with genu valgum. Am J Sports Med 46:1632–1640. 10.1177/0363546518765479 29688749 10.1177/0363546518765479

[22] Tan SHS, Hui SJ, Doshi C et al (2020) The outcomes of distal femoral varus osteotomy in patellofemoral instability: a systematic review and meta-analysis. J Knee Surg 33:504–512. 10.1055/s-0039-1681043 30822786 10.1055/s-0039-1681043

[23] Zhang Z, Cao Y, Song G et al (2021) Derotational femoral osteotomy for treating recurrent patellar dislocation in the presence of increased femoral anteversion: a systematic review. Orthop J Sports Med 9:23259671211057126. 10.1177/23259671211057126 34881342 10.1177/23259671211057126 PMC8647269

[24] Zhang Z, Song G, Li Y et al (2021) Medial patellofemoral ligament reconstruction with or without derotational distal femoral osteotomy in treating recurrent patellar dislocation with increased femoral anteversion: a retrospective comparative study. Am J Sports Med 49:200–206. 10.1177/0363546520968566 33180556 10.1177/0363546520968566

[25] Franciozi CE, Ambra LF, Albertoni LJB et al (2017) Increased femoral anteversion influence over surgically treated recurrent patellar instability patients. arthrosc J arthrosc relat Surg off publ arthrosc assoc. N Am Int Arthrosc Assoc 33:633–640. 10.1016/j.arthro.2016.09.015 10.1016/j.arthro.2016.09.015 27988165

[26] Ammann E, Meier RL, Rutz E et al (2020) Trochleoplasty improves knee flexion angles and quadriceps function during gait only if performed bilaterally. Knee Surg Sports Traumatol Arthrosc Off J ESSKA 28:2067–2076. 10.1007/s00167-020-05906-9 10.1007/s00167-020-05906-9 32130444

[27] Asaeda M, Deie M, Fujita N et al (2016) Knee biomechanics during walking in recurrent lateral patellar dislocation are normalized by 1 year after medial patellofemoral ligament reconstruction. Knee Surg Sports Traumatol Arthrosc Off J ESSKA 24:3254–3261. 10.1007/s00167-016-4040-2 10.1007/s00167-016-4040-2 26869031

[28] Carnesecchi O, Philippot R, Boyer B et al (2016) Recovery of gait pattern after medial patellofemoral ligament reconstruction for objective patellar instability. Knee Surg Sports Traumatol Arthrosc Off J ESSKA 24:123–128. 10.1007/s00167-014-3347-0 10.1007/s00167-014-3347-0 25274090

[29] Strecker W (2006) Planerische analyse Kniegelenknaher Beinachsenabweichungen: I. Deformitäten in der frontalebene. Oper Orthop Traumatol 18:259–272. 10.1007/s00064-006-1175-1 16953350 10.1007/s00064-006-1175-1

[30] Caton JH, Dejour D (2010) Tibial tubercle osteotomy in patello-femoral instability and in patellar height abnormality. Int Orthop 34:305–309. 10.1007/s00264-009-0929-4 20066411 10.1007/s00264-009-0929-4 PMC2899368

[31] Nelitz M, Lippacher S, Reichel H, Dornacher D (2014) Evaluation of trochlear dysplasia using MRI: correlation between the classification system of dejour and objective parameters of trochlear dysplasia. Knee Surg Sports Traumatol Arthrosc Off J ESSKA 22:120–127. 10.1007/s00167-012-2321-y 10.1007/s00167-012-2321-y 23196644

[32] Schneider B, Laubenberger J, Jemlich S et al (1997) Measurement of femoral antetorsion and tibial torsion by magnetic resonance imaging. Br J Radiol 70:575–579. 10.1259/bjr.70.834.9227249 9227249 10.1259/bjr.70.834.9227249

[33] Imhoff FB, Cotic M, Liska F et al (2019) Derotational osteotomy at the distal femur is effective to treat patients with patellar instability. Knee Surg Sports Traumatol Arthrosc Off J ESSKA 27:652–658. 10.1007/s00167-018-5212-z 10.1007/s00167-018-5212-z 30315327

[34] Hinterwimmer S, Minzlaff P, Saier T et al (2014) Biplanar Supracondylar femoral derotation osteotomy for patellofemoral malalignment: the anterior closed-wedge technique. Knee Surg Sports Traumatol Arthrosc Off J ESSKA 22:2518–2521. 10.1007/s00167-014-2993-6 10.1007/s00167-014-2993-6 24748287

[35] Feucht MJ, Mehl J, Forkel P et al (2017) Distal femoral osteotomy using a lateral opening wedge technique. Oper Orthopadie Traumatol 29:320–329. 10.1007/s00064-017-0503-y 10.1007/s00064-017-0503-y 28577210

[36] Fulkerson JP (1983) Anteromedialization of the tibial tuberosity for patellofemoral malalignment. Clin Orthop 177:176–181 6861394

[37] Banke IJ, Kohn LM, Meidinger G et al (2014) Combined trochleoplasty and MPFL reconstruction for treatment of chronic patellofemoral instability: a prospective minimum 2-year follow-up study. Knee Surg Sports Traumatol Arthrosc 22:2591–2598. 10.1007/s00167-013-2603-z 23851967 10.1007/s00167-013-2603-z

[38] Bereiter H, Gautier E (1994) Trochleoplasty as surgical approach for the treatment of recurrent patella instability in patients with trochlear dysplasia of the femur. Arthroskopie 7:281–286

[39] Schöttle PB, Hensler D, Imhoff AB (2010) Anatomical double-bundle MPFL reconstruction with an aperture fixation. Knee Surg Sports Traumatol Arthrosc Off J ESSKA 18:147–151. 10.1007/s00167-009-0868-z 10.1007/s00167-009-0868-z 19593547

[40] Staheli LT, Corbett M, Wyss C, King H (1985) Lower-extremity rotational problems in children. Normal values to guide management. J Bone Joint Surg Am 67:39–47 3968103

[41] Kolowich PA, Paulos LE, Rosenberg TD, Farnsworth S (1990) Lateral release of the patella: indications and contraindications. Am J Sports Med 18:359–365. 10.1177/036354659001800405 2403183 10.1177/036354659001800405

[42] Manske RC, Davies GJ (2016) Examination of the patellofemoral joint. Int J Sports Phys Ther 11:831 27904788 PMC5095938

[43] Graf KH, Tompkins MA, Agel J, Arendt EA (2018) Q-vector measurements: physical examination versus magnetic resonance imaging measurements and their relationship with tibial tubercle–trochlear groove distance. Knee Surg Sports Traumatol Arthrosc 26:697–704. 10.1007/s00167-017-4527-5 28378138 10.1007/s00167-017-4527-5

[44] Gwynne CR, Curran SA (2018) Two-dimensional frontal plane projection angle can identify subgroups of patellofemoral pain patients who demonstrate dynamic knee valgus. Clin Biomech 58:44–48. 10.1016/j.clinbiomech.2018.06.021 10.1016/j.clinbiomech.2018.06.021 30015205

[45] Kibler WB, Press J, Sciascia A (2006) The role of core stability in athletic function. Sports Med 36:189–198. 10.2165/00007256-200636030-00001 16526831 10.2165/00007256-200636030-00001

[46] Livengood AL, DiMattia MA, Uhl TL (2004) Dynamic trendelenburg: single-leg-squat test for gluteus medius strength. Int J Athl Ther Train 9:24–25. 10.1123/att.9.1.24

[47] Light RJ (1971) Measures of response agreement for qualitative data: some generalizations and alternatives. Psychol Bull 76:365–377. 10.1037/h0031643

[48] Landis JR, Koch GG (1977) The measurement of observer agreement for categorical data. Biometrics 33:159–174 843571

[49] Arnold AS, Komallu AV, Delp SL (1997) Internal rotation gait: a compensatory mechanism to restore abduction capacity decreased by bone deformity? Dev Med Child Neurol 39:40–44. 10.1111/j.1469-8749.1997.tb08202.x 9003728 10.1111/j.1469-8749.1997.tb08202.x

[50] Mackay J, Thomason P, Sangeux M et al (2021) The impact of symptomatic femoral neck anteversion and tibial torsion on gait, function and participation in children and adolescents. Gait Posture 86:144–149. 10.1016/j.gaitpost.2021.03.004 33725582 10.1016/j.gaitpost.2021.03.004

[51] Flury A, Jud L, Hoch A et al (2021) Linear influence of distal femur osteotomy on the Q-angle: one degree of varization alters the Q-angle by one degree. Knee Surg Sports Traumatol Arthrosc Off J ESSKA 29:540–545. 10.1007/s00167-020-05970-1 10.1007/s00167-020-05970-1 32274549

[52] Bruderer-Hofstetter M, Fenner V, Payne E et al (2015) Gait deviations and compensations in pediatric patients with increased femoral torsion. J Orthop Res Off Publ Orthop Res Soc 33:155–162. 10.1002/jor.22746 10.1002/jor.22746 25284013

[53] Schranz C, Sperl M, Kraus T et al (2023) Different gait pattern in adolescence with patellofemoral instability. Clin Biomech 108:106067. 10.1016/j.clinbiomech.2023.106067 10.1016/j.clinbiomech.2023.106067 37633176

